# Blockchain-Based Scalable and Tamper-Evident Solution for Registering Energy Data

**DOI:** 10.3390/s19143033

**Published:** 2019-07-10

**Authors:** Claudia Pop, Marcel Antal, Tudor Cioara, Ionut Anghel, David Sera, Ioan Salomie, Giuseppe Raveduto, Denisa Ziu, Vincenzo Croce, Massimo Bertoncini

**Affiliations:** 1 Computer Science Department, Technical University of Cluj-Napoca, Memorandumului 28, 400114 Cluj-Napoca, Romania; 2 R&D Department, Engineering Ingegneria Informatica S.p.A., Via San Martino della Battaglia 56, 00185 Rome, Italy

**Keywords:** Blockchain, imbalances settlement in energy markets, smart energy grid, second tier scalable solution, tamper-evident, off-chain and on-chain energy data storage

## Abstract

Nowadays, it has been recognized that blockchain can provide the technological infrastructure for developing decentralized, secure, and reliable smart energy grid management systems. However, an open issue that slows the adoption of blockchain technology in the energy sector is the low scalability and high processing overhead when dealing with the real-time energy data collected by smart energy meters. Thus, in this paper, we propose a scalable second tier solution which combines the blockchain ledger with distributed queuing systems and NoSQL (Not Only SQL database) databases to allow the registration of energy transactions less frequently on the chain without losing the tamper-evident benefits brought by the blockchain technology. At the same time, we propose a technique for tamper-evident registration of smart meters’ energy data and associated energy transactions using digital fingerprinting which allows the energy transaction to be linked hashed-back on-chain, while the sensors data is stored off-chain. A prototype was implemented using Ethereum and smart contracts for the on-chain components while for the off-chain components we used Cassandra database and RabbitMQ messaging broker. The prototype proved to be effective in managing a settlement of energy imbalances use-case and during the evaluation conducted in simulated environment shows promising results in terms of scalability, throughput, and tampering of energy data sampled by smart energy meters.

## 1. Introduction

The rising shares of intermittent decentralized renewable energy prosumption sources are completely changing the way in which electricity grids are managed to provide electricity to consumers while preserving continuity and security of supply at affordable costs. The smart grid management problems can no longer be efficiently addressed by using centralized management solutions, the need for developing decentralized approaches and architectures are widely recognized. At the same time, the deployment of IoT (Internet of Things) smart energy sensing devices that measure, collect, and communicate energy data has not only created the opportunity for developing rather sophisticated smart grid management services but also generated problems in managing the big amounts of generated data.

Thanks to the increased efficiency of end-user appliances, low-cost photovoltaics, and disruptive technologies such as virtual financial services, decentralized energy networks are rapidly spreading, contributing to low-carbon, sustainable energy systems [1]. The integration of decentralized energy systems at neighborhood scale allows to lower peaks in energy demands on the electrical grid, to reduce the overall consumption [2], and reduce the disparity terms of reliability and cost in rural and remote areas [3]. Thus, future energy systems should be characterized by the key principles of decarbonization, decentralization, and digitalization [4]. 

From our point of view, new designs should be considered for distributed storage and for the validation of energy data as close as possible to the registration point. When addressing the distributed storage, the new solutions should be scalable in terms of data size as well as data throughput. Furthermore, functionalities regarding the data validation and identification of corrupted energy data should be provided in near real-time fashion, in order to ensure a reliable production and consumption matching and a fast-financial settlement. 

Nowadays, it has been recognized that blockchain may provide the needed technological solutions for developing decentralized, secure, and reliable smart grid management systems [2,5,6]. However, even if in terms of reliability and control blockchain is a good solution, being able to decentralize the entire management process of the smart grid, the scalability when integrating the real-time energy data collected by the sensors is still a pressing issue. In some grid management cases, the drawbacks limiting blockchain adoption to energy domain are the extremely high costs for storing data, the high hardware requirements for processing data, and the small transaction throughput especially when dealing with many peers. Smart grid services have diverse requirements in terms of response time which impacts the granularity needed for energy data monitoring and the costs of energy blockchain integration. In the case of ancillary services, as they are usually targeting the safety of the grid, their monitoring requirements are very close to real-time. For example, in Europe [7] the manual frequency restoration reserve features an average response time of 15 min, for Automatic Frequency Restoration the average response time is 5 min, while for Frequency Control Reserve average response time should be 30 s. Anyway, studies have concluded that an energy data sampling rate of one minute is enough to determine power states for electrical loads of most of the regular prosumers [8] and can be successfully used for grid services with more relaxed response time constrains such as, congestion management, unbalances settlement, energy billing, etc. However, even in this case the cost of storing one-minute energy data as transactions in blockchain can be really high. Considering the gas price of 3 Gwei (the safe low gas price [9] of the Ethereum public transaction promising the transaction confirmation in 12 min) and the estimated gas consumed per transaction of 137,131, the cost for one energy transaction is 411,393 Gwei (0.1234179 Euro per transaction at a price of 300 Euros for one Ether). The trading period for the imbalance settlement process generates a cost of 3.70 Euros for each prosumer when storing energy transactions for half an hour. Storing data on the blockchain is very expensive with respect to traditional database systems. For example, in the public Ethereum network to store 1 kB of data one must pay approximately 640 k gas. With an average price for the gas unit of 3 Gwei we end up paying 0.00192 Ether, which is approximately 0.57 Euros at the exchange rate of 300 Euros, resulting in 0.56 million Euros for storing 1 GB of data. Also, by increasing the size of the chain, more hardware resources will be used by the full nodes to store and process the blockchain data. High hardware requirements would lead to a decrease in the number of full nodes available, eventually threatening the reliability of the entire blockchain system which will manage the energy grid. At the same time, nowadays blockchain systems feature a small transaction throughput: Bitcoin allows up to seven transactions [10] per second, Ethereum allows 15 transactions [11] per second, etc. Thus, considering that the smart energy sensors have sampling rates at intervals of a few seconds it is unrealistic and infeasible to register and process energy transactions at similar rates into the blockchain. 

We address the above presented issues by bringing the following novel contributions:

- A scalable second tier solution combining blockchain ledger with distributed queuing systems and NoSQL database to allow the registration of energy transactions less frequently on the chain (e.g., 1 transaction per 30 min) while taking advantage on the high scalability of the NoSQL database for off-chain energy data and of the tamper-proof, provenance tracking and self-enforcing smart contracts benefits brought be the blockchain technology for the on-chain stored one;
- A tamper-evident registration of smart meters’ energy data and associated energy transactions using digital fingerprinting allowing the energy transaction to be linked hashed-back on-chain while the sensors data is stored off-chain;
- Solution validation considering as use-case the settlement of imbalances in power markets, showing its effectiveness in assessing deviations from energy plans and tracking source of that deviation in near real-time while featuring low computational costs and high transactions throughput.

The rest of the paper is organized as following: Section 2 describes the relevant related work in the area of blockchain and energy and solutions addressing the identified scalability problems; Section 3 presents our second tier solution for storing energy transactions and the digital fingerprinting of relevant data while Section 4 presents evaluation results focusing on the scalability improvements and tamper-evident features of our solution. Finally, Section 5 presents conclusions and future work. 

## 2. Related Work

The idea of using blockchain in the energy sector has taken an increasingly large interest in academic research, industry stakeholders, utility companies, and energy decision makers. According to a recent study on 140 blockchain innovation projects and research initiatives [5], in the energy space it is possible to identify eight use-cases: (1) Metering/billing and security; (2) cryptocurrencies, tokens, and investment; (3) decentralized energy trading; (4) green certificates and carbon trading; (5) grid management; (6) IoT, smart devices, automation and asset management; (7) electric e-mobility; and (8) general purpose initiatives and consortia.

The most intuitive—and popular—application of blockchain to the electric power sector is to turn the electricity grid into a peer-to-peer network for prosumers which trade electricity with one another (e.g., buy and/or sell energy generation surplus to neighbors) [12]. In general, the energy surplus is measured by smart meters and then transformed in equivalent energy tokens that can be traded in a marketplace setup at local grid level [13]. Energy tokens or renewable certificates are defined and serve as proof that the electricity has been generated from renewable energy source, to encourage low-carbon and green energy production [14,15,16]. Most state-of-the-art approaches focused on the P2P (Peer to peer) energy market creation [17,18,19] are aiming to demonstrate that blockchain-based intermediary-free energy trading is possible and that is beneficial to both the generators and buyers. Other studies are focused on the optimal management of energy resources by P2P trading in local micro-grids [20].

The use of blockchain technology for the emerging field of IoT [21] and, subsequently, energy efficiency in smart grid is another area of active research [22,23]. Blockchain and self-enforcing smart contracts can facilitate data exchange and machine-to-machine (M2M) communication among energy metering devices. It is estimated that an increasing number of smart devices (20.8 billion) could be connected to the Internet by 2020 [24]. A similar trend is followed by the energy sector where smart meters and ICT equipment are increasingly being adopted [25]. An IoT infrastructure for sharing economy, named Universal Sharing Network (USN), is proposed by slock.it [26], while Oli SharEnergy [27] propose a software platform to enable the optimization of smart grid components, such as single small power plants, prosumers, and storage system. The smart energy grid is formed by ‘energy cells’ (one cell is composed of at least one generation unit and single or multiple loads) which collaborate with each other via blockchain [27]. When the metering infrastructures are integrated with blockchain technology, it is possible to benefit from automated billing in energy services for consumers and distributed generators, which leads to the reduction of administrative costs [28]. Similarly, in Stephant et al. [29] blockchain technology is used for collective self-consumption aiming to certify, validate, and automatically execute transactions between consumers and energy producers via blockchain. Smart meters data is registered into a consortium blockchain and shared with Distribution System Operator (DSO) and energy suppliers to trace energy generation, having a more accurate billing [30]. 

Blockchain may play an important role in the field of grid management, for example, improving the coordination between transmission and distribution system operation or improving the balance of energy supply and demand [31]. The blockchain-based implementation can: Manage the request of balancing power between Transmission System Operator (TSO), Balancing Responsible Party (BRP), DSO, aggregators, and generation units and to enable DSOs to interact with the balancing request process in congestion situations well before the delivery period and not just at the stage when the generation load is ramped up by the aggregator [32]. Furthermore, a solution that provides management and evaluation mechanisms of the prosumers’ activity in demand response programs is presented in Pop et al. [33]. The advent of Electric Vehicles (EV) and e-mobility poses serious energy grid management problems. In this context, the blockchain technology brings several advantages, such as elimination of a centrally managed EV charging infrastructure, fault tolerance, elimination of price-setting, collusion between charging stations, or transport providers. Some researchers propose to integrate blockchain with EV to allow customers to find the cheapest charging station [34] within a previously defined region while preserving their privacy [35,36].

As previously reported, many energy companies are investing in blockchain and IoT as it clearly benefits energy system operations, markets, and consumers [37,38], offering disintermediation and transparency in the energy grid management processes, but most of all, it offers novel solutions for authorizing consumers and small renewable generators to play a more active role in the energy market and monetize their assets [39]. Besides the regulatory framework in the energy sector [40,41], the other aspect that can slow the adoption of blockchain, is the low scalability and high processing overhead. 

To address the scalability challenges of the IoT systems, few solutions have been implemented using different flavors of databases: Centralized databases, NoSQL databases, and distributed databases. Most of the times the database to be chosen for IoT systems is evaluated among existent solutions like MongoDB or Cassandra [42]. Custom implementations [43,44] are proposed to address specific problems like scalability or security. While these solutions provide promising results in terms of storage and throughput, and surpass the promises of a blockchain solution, the trade-offs introduced are represented by lower data integrity and higher susceptibility to failures. The state-of-the-art approaches on energy and storage solutions can be roughly classified under three main categories (see Table 1):

- Firstly, use the blockchain for storing data on-chain, which is extremely costly, and at the same time is inadequate in use-cases where high scalability is required;
- Secondly, use existing distributed databases solutions that are well known for the high scalability and implement some level of decentralized control. However, their shortcomings in terms of Byzantium tolerance and immutability are rendering them unsuitable for specific use-cases where Byzantine attacks can be expected;
- Thirdly, use custom blockchain-based databases such as the BigchainDB [45] that relies on the Tendermint [46] blockchain for transaction broadcasting and consensus, as well as on MongoDB [47] for off-chain storage.

As seen in Table 1, the blockchain databases, such as BigchainDB, seem to be the most suitable solution because they allow for prosumers-defined energy assets to be stored, while ensuring high scalability and a tamper-evident solution (changes cannot be prevented but will be detected). However, the major disadvantage is that it does not provide smart functionality over the registered values. According to the official website of the BigchainDB solution [48], to apply any logic or activity evaluation upon the registered values, another chain providing smart contracts capabilities must be considered that can fetch data from BigchainDB through different mechanisms (e.g., Oracles [49]) and apply custom logic through the execution of smart contracts on the fetched values. 

Our novel hybrid solution addresses the identified disadvantages being based on a symbiosis between distributed databases and blockchain solutions benefiting on the advantages of each: The scalability of distributed databases and the immutability and Byzantine tolerance of the blockchain. Moreover, it offers a scalable solution to the major challenge of integrating an energy monitoring system with the blockchain, since it is unfeasible from a technical and cost perspective to process and store data at the rate provided by the smart energy meters’ sensors (e.g., one energy transaction at every five seconds). 

## 3. Distributed Ledger for Energy Transactions

We propose the implementation of a blockchain-distributed ledger in which energy transactions are generated, registered, and immutably stored in blocks based on the monitored energy data of individual prosumers. The prosumer is modeled as a node of the P2P distributed energy network (i.e., a graph of peer nodes) and will keep a copy of the blockchain ledger which will be automatically updated when new energy transactions will be registered. Other energy players, such as the energy aggregators or the DSO that are interested in micro-grid management, are also registered as network peers. The flow of energy between prosumers will be then represented in the blockchain as transfer energy transactions between two prosumers contracts. To prove the ownership of energy, the prosumer provides the signature over the transfer transaction showing that the energy asset is his/hers, thus authenticating and validating the transfer. Whenever a new prosumer joins the blockchain network, a new account and associated contract is created, and the corresponding node will be connected to a predefined list of seed nodes. The seed nodes will provide the new joined node with information about all the prosumer peers they know about, the process being repeated with the new discovered peers, until the new node builds its own list of peers. 

**Figure 1:**
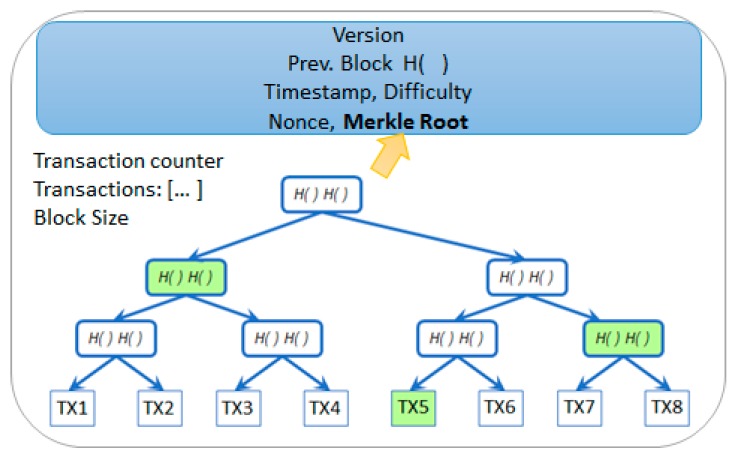
Merkle Tree for storing multiple energy transactions (TX) in a block and Merkle Path example.

To provide better scalability and decrease the length for the chain, multiple transactions (denoted as TX1–TX8) are aggregated in a single block (see Figure 1). Depending on the solution, different approaches have been used in order to keep track and encode the transactions mined in a block. In Bitcoin the transactions of the block are grouped and encoded using Merkle Trees. To support the protocol enhancement, the Ethereum [50] solution uses three different modified Merkle Patricia Tries per block that store key value pairs: State and Storage Trie, Transaction Trie, and Receipt Trie. To build a Merkle Tree, all energy transactions in the block are paired two-by-two and the tree is built from the bottom to the top based on the hashes (H) of these transactions. The tree leaves will contain the transactions while the upper levels will be incrementally constructed by pairing and combining the hashes of two elements from an inferior level until the root is reached. In this way, a binary hash tree is built up to the root. The result is a 32-byte string encoding an entire set of data. Due to its structure, any change that occurs at leaf level, due to tampering with data, will trigger modification up to the root of the tree.

The Merkle Tree Root hash encodes the entire collection of energy transactions that are aggregated in the current block. This hash value is very important because it is responsible to ensure the validity and the integrity of the recorded energy transactions in time. The root hash is added in the header of the block (see Figure 1) and, together with all the other fields in the header, is used to generate the hash of the block. The block hash is further used to identify a block in the entire blockchain. The hash of the block is not stored in the current’s block structure or in the blockchain storage; instead, it is always computed by each node based on the information contained in the header and used as a hash pointer in the following block. The hash of the tree root provides a smaller footprint, an important advantage for the prosumer nodes that do not have enough storage capabilities (i.e., light nodes). Thus, the prosumers associated light nodes will store only the header of the blocks while the actual energy transactions will be stored remotely. The Merkle tree root will provide enough information for light prosumer nodes to be able to check the consistency of the chain. At the same time, light prosumer nodes may interrogate other network full nodes for information to verify if an energy transaction was mined and to identify the block that stores the actual transaction. 

One of the simplest ways to prove that an energy transaction is stored in a block would be to obtain all the transactions of a block and rehash the entire tree to obtain the root hash. If the root hash obtained is the same with the one stored in the header of light prosumer node block, due to the collision free and data binding properties of the hash functions, it would lead to the conclusion that the specific energy transaction was successfully mined. However, this process is not optimal. Since a large number of energy transactions can be included in the block, the verification process is significantly improved by providing only a path instead of the entire set of transactions. For example, consider that the light prosumer node needs to find if energy transaction TX6 was mined or not. It will request a path that proves the membership of this transaction in a specific block as presented in Antonopoulos et al. [50,51] for systems like Bitcoin and Ethereum. This path will contain the hash roots of the subtrees that are not influenced by the hash of the TX6 transaction, marked by green nodes in Figure 1. In this sense, the first hash of the path is the hash of the transaction that was paired with TX6. The hash of the two transactions will be then paired with the second hash from the path, and so on, until reaching the root of the tree. The performance improvement of this approach is considerable, since the node will need to compute only log(N) hashes to prove the membership of an energy transaction. 

Due to the high costs associated with the blockchain processing of data, it is not scalable to register an energy transaction each time new energy data is sampled by the smart metering devices. Thus, we developed a scalable second tier approach allowing for energy transactions to be registered less frequently on the chain (e.g., one transaction per half hour) while not losing any of the benefits brought by the blockchain technology. This approach is detailed below.

### 3.1. Scalable Second Tier Solution

In our proposed hybrid solution, the real-time energy data collected from IoT metering devices during a predefined time interval is hashed-linked back at prosumer level in the order in which it was sampled and then is stored off-chain in a distributed database. As a result, a single energy transaction is created and signed for the entire interval by the prosumer. The transaction will be published on blockchain, containing the average energy value registered and the generated hash fingerprint. 

On blockchain, the registered average energy value will be used for further validation and business logic assessment [33], while the digital fingerprint transaction will be linked hashed-back with the digital fingerprints of energy transactions generated for previous time intervals. Whenever historical data is requested, the digital fingerprint of the off-chain stored data will be computed and checked against the on-chain registered fingerprint. In case the hashes coincide, it is concluded that the off-chain real-time registered data has not been tampered. Otherwise, further inquiries can be made to detect the exact interval where the data has been modified, thus leading to a tamper-evident system, where no changes can go unnoticed.

**Figure 2:**
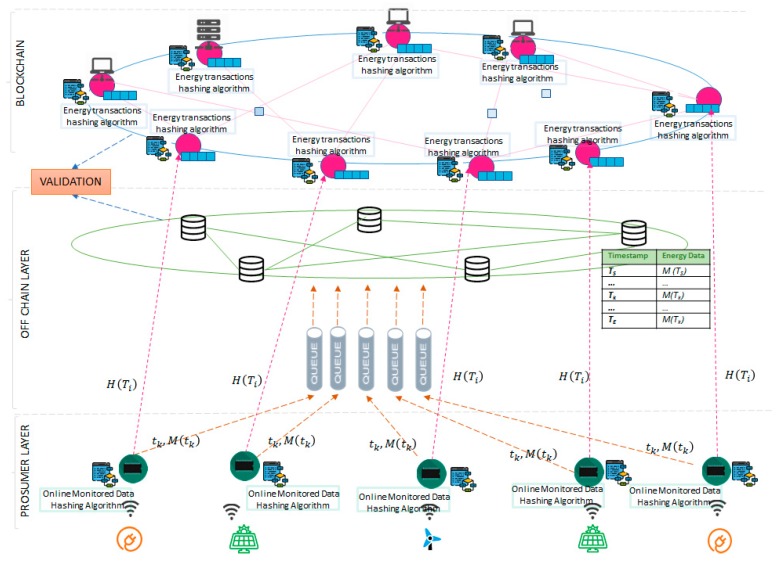
Proposed second tier solution for energy data storage.

Figure 2 presents the layered architecture of our proposed solution:

- On the prosumer layer the energy data is fetched from sensors that are characterized by a device identifier and a measurement type, the hashing algorithms are executed and the connections to store the raw values are implemented;
- On the off-chain layer scalable storage capabilities are provided and used for the real-time data received from the sensors. A queue-based asynchronous messaging system is used to ensure the system operation even in case of fluctuations in the data sampling rate;
- The blockchain layer stores the energy transactions in blocks.

Let us consider an energy metering device of a prosumer that provides monitored data over a time interval P. We split interval P in N smaller disjoint intervals $T_{i}$, where each interval $T_{i}$ is delimited by a start time and an end time $T_{i}=\left(T_{S}^{i},\;T_{E}^{i}\right]$:

(1) $$P=\overset{N}{\underset{i=1}{\cup }}T_{i}and\overset{N}{\underset{i=1}{\cap }}T_{i}=\emptyset$$

In each time interval $T_{i}$ there can be L ordered discrete timestamps in which sensors are sampling new data:

(2) $$T_{i}=\{t_{k}|t_{k}<t_{k+1},\;\forall k=0..L-1\}$$

We denote the monitored value sent by a sensor at a timestamp $t_{k}$ as $M\left(t_{k}\right)$. From the sensor level, the monitored data value $M\left(t_{k}\right)$ is sent to the edge device at timestamp $t_{k}$. The edge device will forward this information directly to the asynchronous messaging system that stores the new sample data in the off-chain storage system in a sequential manner. At edge device level we defined an online hashing algorithm that for each interval $T_{i}$ will compute the digital fingerprint of all monitored data received from the sensor at each discrete timestamp from that interval. At the end of interval $T_{i}$, the edge device will sign and register the digital fingerprint of the monitored data on blockchain, thus ensuring an immutable log of this value. The blockchain will implement a decentralized storage algorithm that is responsible to store and compute a hash of period P, composed of all the digital fingerprints received for each interval $T_{i}$.

### 3.2. Digital Fingerprinting Energy Data 

We defined an online algorithm (see Algorithm 1) that aims at computing the hash of the raw monitored energy values as they are sampled for each discrete timestamp over an interval $T_{i}.$ Thus, the inputs of the algorithm are the monitored value at each timestamp $t_{k}$ and a seed hash (HASH_SEED_) that is uniquely generated for each prosumer, representing the genesis hash value for the linked back data structure. The outputs expected to be returned by the algorithm are the hash representing the digital fingerprint of all the monitored energy data values over the period $T_{i}$ and the average energy value over the entire interval. 

The first step of the algorithm at line 1 is to initialize the hash of the period $T_{i}$ with the value of the seed. For each new energy monitored data $M\left(t_{k}\right)$ at timestamp $t_{k}$ in the interval $T_{i}$ (line 2 and 3), the hash $H\left(t_{k}\right)$. will be computed (line 3) by hashing the current value, with the timestamp and the previous hash registered at $t_{k-1}$ (for time $t_{1}$, the previous hash is $H\left(T_{S}^{i}\right)$—the seed hash). At the same time the average energy is calculated (line 4, 6) so that at the end of the period $T_{i}$ (line 7) the average will be returned together with the fingerprint $H_{t_{k}}$, that is the hashed-linked back values over the entire period $T_{i}$. 


**Algorithm 1: Hashing Energy Data Collected by Energy Meter on The Edge Device.**
***INPUT:***$T_{i}$*,*$M\left(t_{k}\right)$*,*$HASH_{SEED}$ ***OUTPUT:***$\;Energy_{Transaction}\langle H\left(T_{E}^{i}\right),AVG_{Energy}\left(T_{i}\right)\rangle$ ***Begin*** 1. $H\left(T_{S}^{i}\right)=HASH_{SEED}$ 2.
$foreach\left(M\left(t_{k}\right),T_{S}^{i}<t_{k}\leq T_{E}^{i}\right)\;do$ 3.
$H\left(t_{k}\right)=HASH\left(M\left(t_{k}\right),t_{k},H\left(t_{k-1}\right)\right)$ 4.
$SUM_{Energy}\left(t_{k}\right)=SUM_{Energy}\left(t_{k-1}\right)+M\left(t_{k}\right)$, count++ 5.
endforeach 6.
$AVG_{Energy}\left(T_{i}\right)=\;\frac{SUM_{Energy}\left(t_{k}\right)}{count}$ 7.
$return\;Energy_{Transaction}\langle H\left(T_{E}^{i}\right),AVG_{Energy}\left(T_{i}\right)\rangle$ ***End***

**Figure 3:**
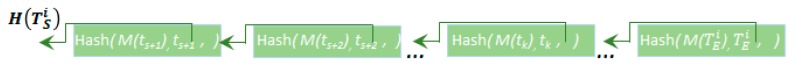
Hashed linked back monitored values.

The final hash obtained at the end of the interval is a digital fingerprint of all the values. The hashed-linked structure is chosen to accommodate the seriality of the data received from the sensors as well as any fluctuations in the sample rate. Since at each point in time the only value required is the previous hash (as depicted in Figure 3), this structure can be easily used by devices with very low hardware specifications, as opposed to a Merkle-Tree structure, where previous data must be kept in memory in order to compute pair-wise hashes of data. 

During time interval $T_{i}$ each monitored energy value is sent to a distributed NoSQL database which can easily handle large amounts of data. However, at the end of the time interval $T_{i},$ only the digital fingerprint $H_{t_{k}}$ is registered and saved on-chain together with the average of the monitored energy values $AVG_{Energy}$. Since this point forward, the immutability of the fingerprint is ensured by the blockchain. Furthermore, the immutability of the actual data $M\left(t_{k}\right),\;t_{k}$ is ensured in the sense that any tampering will not go unnoticed, thus rendering the off-chain data tamper-evident and the on-chain data tamper-proof.

For fingerprinting and storing on-chain energy the transactions a second hashed-linked back structure is defined and used. From the transactional throughput point of view, depending on the chain specifications and on the number of devices installed, the granularity of interval $T_{i}$ should be set such that the chain is able to efficiently process the entire number of transactions received from the network. From the storage point of view, since during the entire period P, there will be N digital fingerprints specific for each interval $T_{i}$, the storage space would increase proportionally with N. Thus, we defined a second hashed-linked back structure (see Algorithm 2) to keep by hash-linking back all the digital fingerprints $H\left(T_{i}\right)$ of all intervals $T_{i}$. The input of the algorithm consists in the sampling number of intervals, N, that the period P is split in, the current interval generated energy transaction, and the seed hash $HASH_{SEED}$**,** required for initializing the hash of the period P (line 1). For each new interval, i registered before the end of the period (line 2 and 3), the hash $H^{T_{i}}$ is computed as the hash of the current interval energy transaction hashed together with the hash of the previous interval $H^{T_{i-1}}$ (for interval $T_{1}$ the previous hash is $H^{T_{0}}$—the seed hash). The output of the algorithm is the hash value generated for the entire interval P.


**Algorithm 2: On-Chain Linked Back Energy Transactions Hashing.**
***INPUT:****N,*$Energy_{Transaction}\langle H\left(T_{i}\right),AVG_{Energy}\left(T_{i}\right)\rangle ,$$HASH_{SEED}$ ***OUTPUT:***$H_{P}$ ***Begin*** 1. $H^{T_{0}}=HASH_{SEED}$ 2.
foreach (0<i≤N) do 3.
$H^{T_{i}}=H\left(Energy_{Transaction}\langle H\left(T_{i}\right),AVG_{Energy}\left(T_{i}\right)\rangle ,H^{T_{i-1}}\right)$ 4.
endforeach 5.
$return\;H_{P}$ ***End***

The advantage of our proposed approach is that by the end of period P, one fixed-length hash will depict the digital fingerprint of energy data acquired during the entire period regardless of the number of registered intervals N. 

**Figure 4:**
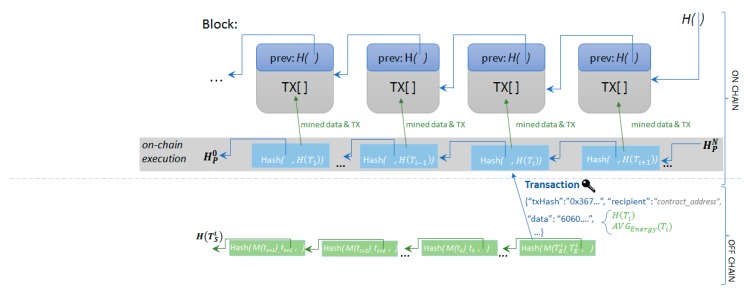
Hashed linked back on-chain energy transactions.

Each hash corresponding for a time interval was mined in a block as depicted in Figure 4 and validated by the network nodes rendering it immutable once enough blocks were mined on top of it. 

**Figure 5:**
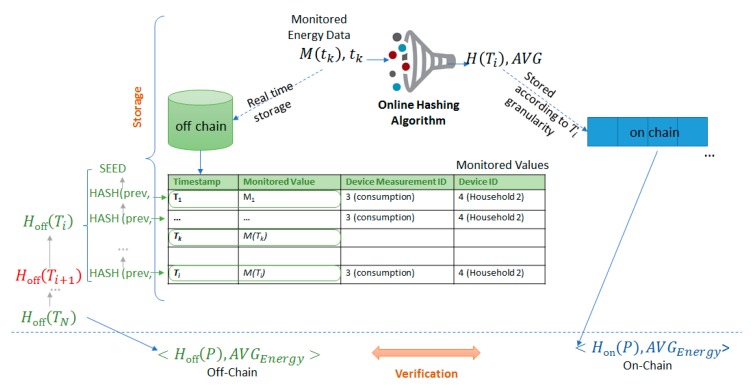
Off-chain energy transactions data validation mechanism.

When historical monitored energy data values need to be retrieved for a period P, all the stored values are validated against the data fingerprints registered on-chain. All the values that are fetched from the database are passed through the algorithms presented in an offline manner, resulting the digital fingerprint of the data, $H_{P}^{off}$ over the time period P. Since the chain already keeps for each period P a tamper-proof fingerprint, $H_{P}^{on}$, considering that the data stored off-chain has not been tampered with, $H_{P}^{off}$ will be equal to $H_{P}^{on}$ as depicted in Figure 5. 

Any changes of monitored energy values or of the associated timestamps will be detected by the algorithm since any modification will render a completely different hash of the data. In case an inequality between the hashes is detected, further inquiries are made to detect the exact interval $T_{i}$ that has been tampered with. Thus, a tradeoff must be considered for choosing the granularity of $T_{i}$ for which the digital fingerprint is registered on-chain. A high sampling rate is beneficial to narrow as much as possible the subset of monitored data that contains the unreliable tampered value, but at the same time could affect the ledger’s scalability since the blockchain can handle a limited throughput. 

## 4. Evaluation Results 

We have considered the settlement of imbalances in power markets as evaluation use-case. Nowadays, this process features significant delays (i.e., a few months) due to latency in energy volumes actualization, reconciliation, and confirmation [5]. Existing literature pin points the blockchain as potential technology for reducing the delays to a minimum. The use of smart contracts has the potential of tracking in a near real-time fashion (i.e., half an hour in our case which is the trading period in the imbalance settlement process [52]) the identity of the prosumer generating the imbalance getting the billing process closer to the real-time. However, the main drawback limiting the technology adoption is the high costs and low scalability of energy metering devices integration with the blockchain distributed ledger [53]. 

**Figure 6:**
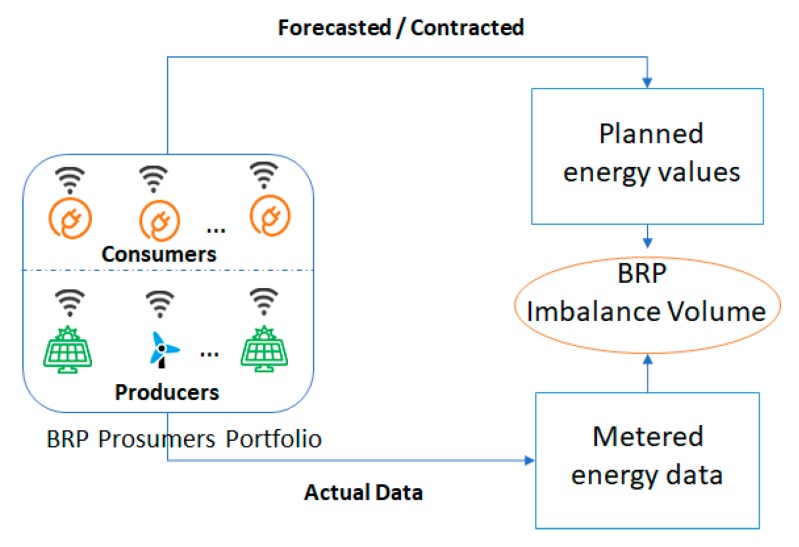
Imbalances settlement process use-case.

In our evaluation, we aim to show how our second tier technical solution could be used for implementing the settlement of imbalances process with low latency and high throughput of transactions processing while benefiting on tamper-evident features and smart contracts-based settlement. The implemented use-case is described in Figure 6.

We have considered a Balance Responsible Party (BRP) which has the responsibility for balancing for a portfolio of 12 prosumers (i.e., 6 producers and 6 consumers). The BRP obligation is to submit energy generation/production schedules to the TSO and it is financially accountable for deviations from those programs [54]. The prosumers energy behavior is simulated leveraging on various energy profiles. The energy profiles of the consumers are taken from a data set provided by Grewal-Carr et al. [55] containing 12-month of minute by minute average power consumption values, while the generation profiles were synthetically generated with the same sampling frequency feature, considering different types of energy production (e.g., wind, solar, etc.). 

The second tier energy storage solution was implemented using Ethereum and Solidity [56] for the on-chain components, while for the off-chain components we used Cassandra and RabbitMQ [57]. The on-chain implementation is enforced by smart contracts that are responsible to process all the digital fingerprints received from energy metering devices associated with the prosumers in the BRP portfolio. 

For evaluation purposes we considered a setup in which P is set for 12 h, while for $T_{i}$ interval we considered a granularity to a half an hour, the energy data being sampled by the energy meters at every minute:

(3) $$T_{i}=\{t_{k}|t_{k}<t_{k+1},\;\forall k=1..30\}\;$$

Thus, energy transactions associated with the consumption and generation of modeled prosumers in the BRP portfolio were published on-chain at the end of each half an hour. 

### 4.1. Tracking of Energy Imbalances

**Figure 7:**
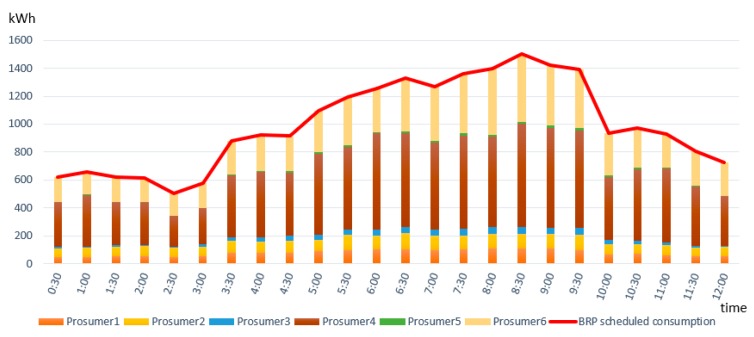
Balancing Responsible Party (BRP)’s scheduled energy plan in relation with enrolled prosumers energy consumption.

We evaluated the effectiveness of our solution to detect the imbalances in terms of deviations between BRP submitted energy plans and the actual delivery energy of prosumers from its portfolio. We implemented and deployed smart contracts allowing to assess at each half an hour the deviations encountered at the level of the BRP and also to track the identity of the responsible prosumer. Figure 7 presents the energy consumption plan constructed by the Balancing Responsible Party (BRP), the values being split on each consumer from its portfolio. 

**Figure 8:**
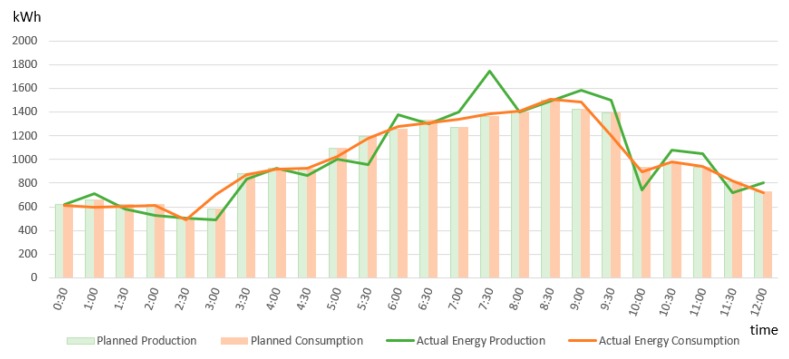
BRP’s total planed vs actual energy consumption.

In our simulation scenario we considered that at certain time frames the actual delivery of some prosumers does not match the plan generating deviations for which the BRP will be financially accountable (see Figure 8).

Each prosumer’s activity is tracked and validated at each half an hour against the plan by means of the smart contracts deployed on-chain. The corresponding smart contracts were triggered by new energy transactions registered in the distributed ledger every half an hour and evaluated the difference between the planed energy consumption and the actual monitored one (as shown by monitored energy transactions registered in the distributed ledger). 

**Figure 9:**
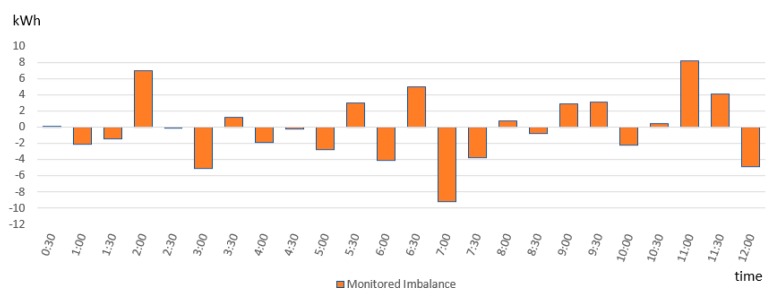
Tracking the deviations of Prosumer1 from energy plan.

Figure 9 shows the imbalances calculated for Prosumer1 where negative values represent the downward regulation (i.e., prosumer’s consumption is higher than anticipated) and positive values represent the upward regulation (i.e., prosumer’s consumption is lower than anticipated) that has to be activated to restore the balance. Each time the imbalances pass the threshold minimum allowed, the BRP was penalized for the imbalance created (for example hours 2, 7, and 11).

**Figure 10:**
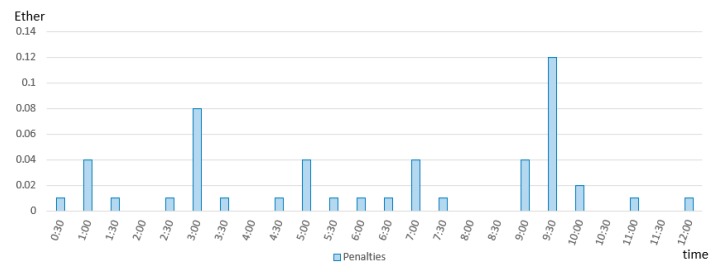
BRP’s financial payment due to registered energy imbalances.

The imbalances generated by the prosumers were further reported to the BRP which may take advantage of the blockchain smart contracts to achieve an internal balancing between generation and production in real-time. For financial settlement we considered the price for the electricity of 0.2 Euro per kWh [58] and the price of Ether of 330 Euro, resulting in a reference price of 606 Gwei per Wh. Considering this, the results presented in Figure 10 depict the financial fees that must be paid by BRP as a consequence of its energy imbalances monitored in near real-time.

### 4.2. Scalability and Tamper-Evident Feature

First, we evaluated the transaction scalability and throughput to show: (i) The feasibility of deploying such architecture in a large network of energy prosumers and (ii) the low cost associated with energy transactions processing. The obtained results described below show the potential of our second tier solution in addressing the latency and costs drawbacks identified in state-of-the-art literature as limiters for adopting the blockchain technology to manage the imbalances settlement process.

Table 2 presents the average throughput and response time results of the proposed second tier solution. We considered the response time as corresponding to the time elapsed from the moment the edge device published the energy value to the point the queue acknowledged the request.

Our solution features a throughput of up to 50,000 transactions per second, providing significant improvement in terms of scalability, while at the same time ensuring the tamper-proofing of data. 

**Figure 11:**
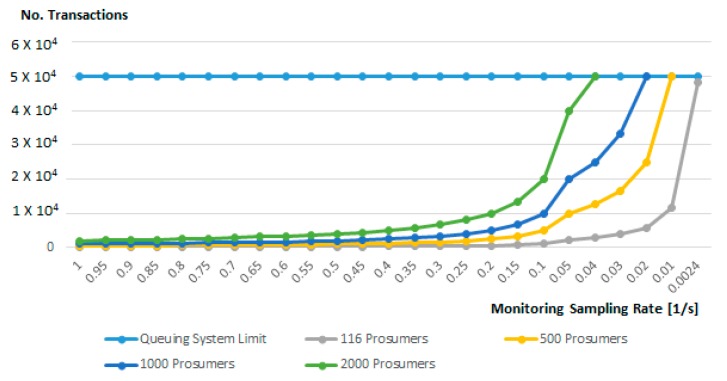
Off-chain monitoring sampling rate and the number of prosumers.

Figure 11 shows the relation between the average monitoring sampling rate, $t_{k}$, and the number of energy transactions that can be processed by the queuing system. As it can be seen for up to 2000 prosumers, an average monitoring sampling rate of five transactions per second is feasible and does not lead to congestion of the queue messaging system.

**Figure 12:**
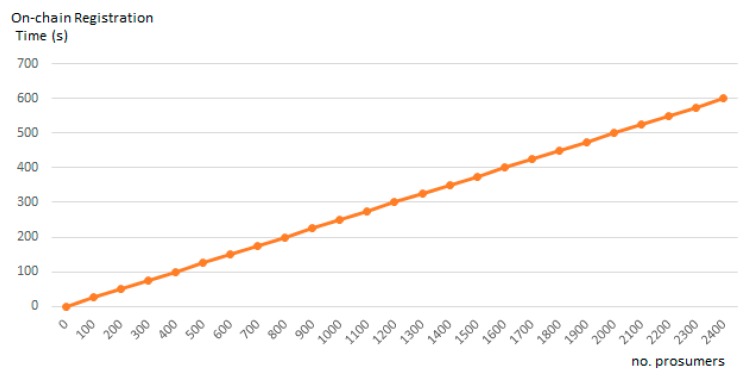
On-chain energy transactions registration time interval and the number of prosumers.

Figure 12 shows the relation between the minimum energy transaction time interval considered $T_{i}$ and the number of prosumers in the grid. As it can be seen, our solution scales are linearly. In case of managing a grid of approximately 2000 prosumers, the minimum interval $T_{i}$ for efficiently register energy transactions is of approximately nine minutes. 

Second, we evaluated the tamper-evident feature of our solution by estimating the impact of tampering the smart meter collected energy data on the transaction registration and on the accuracy of energy forecasting process which have these data as input. In this way, we show how our solution is addressing one of the ways the BRPs could influence their imbalance volume: Less accurate forecasting. 

To evaluate the tamper-evident feature of our solution, we altered the input energy data set considered with a random data tampering pattern and investigated the impact on the value of energy transactions registered on-chain and on energy forecasting processes. We considered a salt-and-pepper noise [59] overlapped on the monitored energy data from smart meters:

(4) $$M{\left(t_{k}\right)}^{'}=M\left(t_{k}\right)+Z\left(t_{k}\right)$$

The noise model is defined considering a probability density function $P_{D}$, a threshold $S_{P}$, and a maximum allowed noise value $Noise_{MAX}$:

(5) $$Z\left(t_{k}\right)=\{\begin{array}{c} Noise_{MAX}\times P_{D}\left(Z\right),\;\;if\;P_{D}\left(Z\right)\leq S_{P}\\ 0,\;\;if\;P_{D}\left(Z\right)>S_{P} \end{array}$$

**Figure 13:**
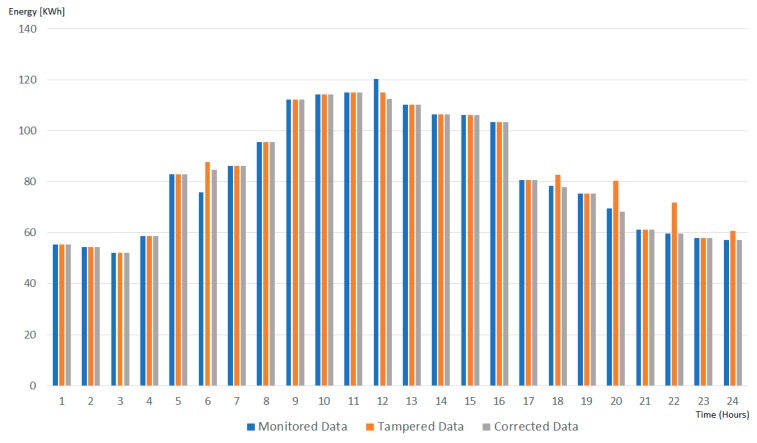
Energy transactions stored on-chain on hourly basis.

Figure 13 presents comparatively the impact of tampering the monitored energy data onto the energy transactions stored on-chain in three cases: No tampering, data tampered, data cleaned using our tampered evident solution. As it can be seen, our solution is managing to identify all the data tampering noise (hours 6, 12, 18, 20, 22, and 24), adjust them, reducing their impact on the energy transactions values registered on-chain (less than 0.75% compared with the ones generated using monitored data).

BRPs use forecasting solutions to estimate the prosumers energy consumption and production considering on historical energy data sampled by IoT energy meters in order to create and submit accurate energy programs. This kind of data that can be easily tampered influencing the accuracy of prediction processes. As already presented in Section 3.2, our solution can identify, through digital fingerprinting, the timeslots with tampered energy data and remove it. Next, we investigated the impact of this process on the energy forecasting accuracy. 

We considered Multi-Layer Perceptron (MLP) based algorithm [60] to predict the energy demand for the next 24 h of a set of energy consumers having a month of monitored energy data. As input features, we have used six days of registered energy transactions with a granularity of one hour while as output we had generated 24 values one for each hour of the day ahead. Same noise models as before were used to tamper the input data of the forecasting process. We used our solution that was capable of identifying the interval $T_{i}$ where data was tampered, and replaced the anomalous data with the average of its neighbors before feeding it to the prediction algorithm. 

The results from Table 3 show the MAPE (Mean Absolute Percentage Error) [60] of the predictions over the test data in three cases: (i) The data is un-tampered; (ii) the data is tampered using a random noise; (iii) the data is tampered using noise, the malicious data is identified by our solution, and the data is replaced using interpolation with its neighbors. As it can be seen, by adding a random tampered data with a probability increasing from 10% occurrence to 40% occurrence, the MAPE of prediction process having as input the data corrected using our solution, is closed to the one when the actual monitored data is considered. Moreover, the MAPE is improved in average with 30% compared with the one obtained on tampered data. 

**Figure 14:**
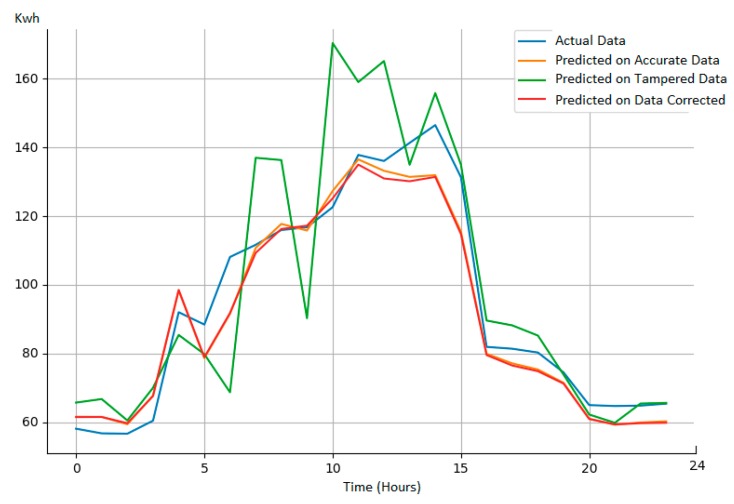
Forecasting process result details on various input data.

Details on the forecasted process results for a specific day having the probability of input data tampering of 40% are presented in Figure 14. One can notice that the forecasted energy curve on data corrected by our solution follows closely the one generated using accurate data. 

### 4.3. Discussion on Other Approaches 

There are second tier solutions in literature which are appreciated for low cost and high transaction throughput, the most notable being the payment channels and the state channels (as a generalized version of the lightning network proposed by Poon et al. [61] and the Raiden Network [62] that considers state updates and not only payments on the off-chain channels). An opening transaction is deployed on-chain, providing the initial setup of an off-chain channel between two network participants. After the channel opening, many transfers (represented by transactions signed by the issuer that can at any point be returned on-chain for settlement or in case of a dispute) can be securely performed among the two participants ensured by hash-time locks that prevent any malicious activity of the parties involved. The cost of this off-chain transfers is insignificant in case that a direct channel is opened between two parties. If an intermediate off-chain node (hop) is required to redirect the message, then the cost can slightly increase, the hop can require a small fee per transfer. The actual value (state or financial assets) cannot be acted upon or used on-chain, until the channel is closed and the state of the blockchain (or balance) is updated considering the latest off-chain transaction. Upon returning on-chain, another two transactions need to be issued (the channel closing and settlement transaction), with the clear benefit of issuing three transactions instead of the N transactions that would be required otherwise. 

However, the off-chain mechanism scalability has a clear benefit as long as the participants are not required to return frequently to the chain and settle the state like in the case of our energy use-case where evaluation against a contracted energy plan, tracking of imbalances, market settlements, is conducted on-chain every half an hour. In this case the transaction costs are significantly higher due to the repeated opening/closing/settlement operations that each require an on-chain transaction leading to a more than triple total transactional cost. Table 4 presents comparatively the costs for energy transactions processing in our unbalancing settlement scenario for two implementation cases, Raiden [62] for payment channels and our second tier solution. 

Furthermore, if the intermediate messages that are transferred on channels off-chain are required for future analysis, forecasting, etc., a local database solution needs to be considered to store this data, similarly with our proposed solution in order to prevent data tampering. A message storage solution is also planned by Raiden to use a Matrix [63] server to keep the message history sent between peers.

The private blockchain could be potential solutions in our problem case as they are described in the literature as featuring high scalability and low transactional costs. While a public blockchain provides fully-decentralization allowing each peer of the energy grid system to provide its contribution to the consistency and security of the chain, the private ones consider consensus algorithms that tend to privilege a set of peer nodes that can have a higher level of control than the regular ones [64]. Furthermore, in this case there is a trade-off on transaction throughput and a number of peer nodes that affect the scalability of the private blockchain solutions. Public chains like Ethereum have set a mining time limit in order to accommodate with the large number of peer nodes and ensure successful communication and synchronization among these nodes. In Decker et al. [65], authors evaluate the propagation time of a block in the Bitcoin system, concluding that it takes an average of 12.6 s for the block to reach 95% of the nodes, and each additional kilobyte in size can cost up to 80 ms delay. If private chains are required to scale to large energy network topologies, the system can encounter similar problems. Similarly, according to an evaluation made in Vukolić [66] even in private deployments a higher number of peer nodes is having a negative impact upon the transaction throughput in the network.

For evaluation, we considered a private Ethereum blockchain setup having Proof-of-Authority as consensus algorithm and the instant-seal setup provided by the Parity client. The latter is considered the best-case scenario setup in terms of scalability since the blocks are instantly sealed and considered correct. The default mining time is set to 15 s, while the gas limit is 8,000,000 gas per block comparable with the public network’s one. In the private setup of the Ethereum chain obtained, the gas consumed per each monitoring transaction is 137,131. The number of energy transactions stored in each block is computed by dividing the block gas limit with the gas consumed by a transaction that registers the energy value (i.e., approximately 58 monitoring transactions can be registered per block).

In Table 5 the response time storing one transaction is computed. In case of the private blockchain setup, this number corresponds to the seconds elapsed from the point the edge device publishes the transaction on the network to the time that a transaction receipt is returned as a result of it being sealed in a block. For the Proof-of-Authority, we used the recommended time of 15 s per block, time in which the network to be able to synchronize and to ensure a correct execution of the protocol, it results in a rate of four transactions per second. An additional constraint considered is that no two energy transactions in a block can have the same pair of sender–receiver addresses. Thus, the on-chain monitoring rate for the system is of minimum 15 s considering a minimum number of 58 prosumers that register their value in each block. From this value, the on-chain monitoring rate should be further increased to accommodate a larger number of prosumers to avoid congestions on-chain. Considering an Instant-Seal setup, where no mining is performed and the transactions are instantly sealed in a block by the node, the transaction rate increases up to 116 transactions per second. Compared to our second tier solution, the results obtained show a lower transactional throughput due to the set constraints in terms of gas per block limit and block mining time (in case of Proof-of-Authority). These values could be potentially optimized, but the restrictions imposed by large networks should be considered [63] since a smart grid network deployment needs to deal with a large number of peer nodes.

## 5. Conclusions

In this paper, we presented a scalable second tier solution combining blockchain ledger with distributed querying systems and NoSQL database to allow the registration of energy transactions less frequently on the chain. In this way, we managed to address the blockchain scalability and processing overhead problems which appear when dealing with energy data frequently sampled from smart meters. Our solution is benefiting on the high scalability of the NoSQL database to store energy monitored data off-chain while keeping the tamper-proof, provenance tracking, and self-enforcing smart contracts benefits for the energy transactions stored on-chain. To achieve this, we propose a tamper-evident solution for registering smart meters’ energy data based on digital fingerprinting, which allows the less frequent registered energy transactions to be linked hashed-back on-chain. We considered the settlement of imbalances in power markets as evaluation use-case to show our approach effectiveness in assessing the potential unbalances, tracking and identifying the source paving the way for bringing the financial settlement process closer to the real-time. The results are also promising in terms of response time and transaction throughput, while the tamper-evident solution is able to identify the modified monitored data reducing its impact on registered energy transaction to less than 0.75% while the impact on the energy forecasting processes to less than 5%. 

## Figures and Tables

**Table 1:** State-of-the-art energy data storage solutions.

	Distributed Database	On-Chain Data	Blockchain Database	Our Second Tier Solution
**Immutability**	no	Tamper-proof	Tamper-evident	Tamper-evident
**Decentralized Control**	yes	yes	yes	yes
**Byzantine Tolerant**	no	yes	yes	yes
**Storage Scalability**	high	low	high	high
**Costly (** **public network)**	no	yes	yes	medium
**Smart Contract**	no	yes	no	yes (for on-chain transactions)

**Table 2:** Evaluation of the second tier solution throughput.

Properties	Second Tier Energy Data Storage
**Response Time**	0.002 s
**Throughput**	50,000 tx/sec

**Table 3:** MLP prediction accuracy on tampered energy data.

Probability of Having Tampered Monitored Data as Input	MAPE Values for		
	Forecasting on Monitored Data	Forecasting on Tampered Data	Forecasting on Data Corrected by Our Solution
10%	5.15%	6.90%	5.41%
20%	5.15%	7.56%	5.55%
30%	5.15%	7.72%	5.81%
40%	5.15%	8.61%	6.12%

**Table 4:** Energy transaction processing costs in gas: Payment channels vs our second tier solution.

Raiden for Payment Channels		Proposed Second Tier Solution
**Channel Opening**	113,807	137,131
**Channel Closing**	146,529	
**Channel Settlement**	192,446 (2—required for each participant)	

**Table 5:** Private blockchain setup results.

	Private Setup of Ethereum	
	Proof-of-Authority	Instant-Seal
**Storage Response Time**	15 s	0.5 s
**No. Transactions/Second**	~4 tx/sec	~116 tx/sec
